# Factors associated with bystander behaviors of Korean youth in school bullying situations: A cross-sectional study

**DOI:** 10.1097/MD.0000000000007757

**Published:** 2017-08-11

**Authors:** Seung Ae Yang, Dong Hee Kim

**Affiliations:** College of Nursing, Sungshin University, Dobong-ro, Kangbuk-gu, Seoul, Korea.

**Keywords:** adolescent, bullying, bystander, school

## Abstract

The behaviors of bystanders can have important effects on their peers. The aim of this study was to identify psychosocial and contextual factors associated with 3 types of bystander behavior (bully followers, outsiders, and defenders of victims) among Korean youth. A descriptive and cross-sectional study was conducted among 416 7th and 8th-grade students from 1 middle school in Korea. The Rosenberg Self Esteem Scale, the Korean version of the Social Problem-Solving Inventory, and measurements of relationships with friends and teachers, empathy, concerns about being bullied, attitudes toward bullying, and bystander behaviors were all used in the assessment. Empathy, relationship of teachers, attitudes toward bullying, and concerns about being bullied were significantly associated with all 3 types of bystanders’ behaviors. Although, self-esteem, social problem solving ability were significantly associated with just defender of victim behaviors. These results suggest that several significant factors to cultivate constructive bystander behaviors should be considered to develop effective antibullying intervention.

## 1 Introduction

School bullying is the most common type of school violence and is increasingly recognized as a major social problem challenging healthy development that has long-lasting negative consequences for children and adolescents.^[1]^ The social dynamics of bullying, and in most school bullying situations, other students not directly involved as bullies or victims are present as bystanders. Bullying is influenced by the interactions among bullies, victims, and bystanders.^[2,3]^ Students by spending time together reciprocate each other's behavior and reinforce one another's acts. Previous studies have shown that a larger peer group of bystanders may either exacerbate or attenuate bullying behavior on social dynamics.^[4,5]^ Bystanders can play an active role by repeatedly and indirectly participate in the victimization process as an individual or a group in a social system. Bystanding may either facilitate or ameliorate victimization, and the bystander is propelled into the role by dint of his or her interaction with the victim and victimizer, and ongoing interaction can be activated in a helpful or harmful direction.

However, prior bullying studies focus on victims and bullies, with bystanders treated as either nonexistent or irrelevant. Despite numerous empirical studies on bullies and victims, critical gaps exist in understanding the group processes involved in bullying. We have for too long focused upon the bystander as an individual who observes but does not invest in the situation he or she witness.

Previous studies suggest different participant roles bystanders may have in the bullying process.^[6,7]^ Seo^[8]^ identified 3 different types of bystander based on Salmivalli and colleagues’ categorization in a Korean bullying situation; each of these types of bystander has a very particular role and investment. The bully followers assist or reinforce the bully's actions by encouraging him or her, or by laughing at the victim's plight. The outsiders attempt to keep away from any and all altercations. The defenders of victims support the victim either by trying to stop episodes of bullying or by offering support and friendship to him or her. Salmivalli bystander behavior types included 4 groups: assistants, reinforcers, outsiders, and defenders, while Seo^[8]^ suggested that the assistant role and reinforcer role should be placed in a single category, “bully-followers.” This 3-factor model of bystander behavior in bullying was also explored via confirmatory factor analysis by Thornberg and Jungert^[9]^ in a Swedish sample (with the labels “pro-bullying,” “outsider behavior,” and “defending”).

Given that bullying is a social dynamic, a bystander's potential influence is important. A few studies have indicated that variability in bullying can be partially explained by the prevalence of bystander behaviors.^[10–12]^ It is necessary to emphasize and understand bystander roles in bullying studies. Specifically, the investigation of bystander behavior and its associated factors will be useful in identifying intervention strategies for decreasing bullying because their behavior might be easier to change than the behavior of aggressive bullies and as a consequence, diminish the social rewards associated with bullying.^[4,13]^

To understand adolescent behavior in social situations, particularly stressful situations such as bullying, we need to explain various influential psychosocial factors. Psychosocial factors are closely related to coping efforts and the behavior of adolescents when facing social stimuli.^[14]^ Positive or negative self-cognition in addition to cognitive factors such as self-esteem, empathy, and social problem-solving ability contributes to behavior in bullying situations.^[9,15,16]^ Additionally, understanding bullying as a group phenomenon implies that social relationships also help explain bullying. Social relationships in school with peers or teachers must be taken into consideration when exploring bystander behaviors in bullying situations in the social context, as bullying serves to organize implicit power hierarchies in schools.^[17,18]^

Also, to understand student's behaviors in bullying situation, contextual factors such as emotion or attitude are revealed in bullying context must be considered. It is particularly important to examine to attitudes toward bullying and concern about being bullied to understand the mental process that eventually guides behavioral responses to social stimuli such as bullying situations.^[14,19]^ Concern about being bullied has been directly linked to adolescent responses to bullying situations.^[8,16,20,21]^

Psychosocial and contextual factors like the above are essential factors to apply to intervention effectively. Identifying factors that influence bullying behavior would be provide some direction for the development of preventive and intervention programs to decrease bullying. Similarly, it is important to find factors affecting each type of bystander behavior. However, there is little information available regarding what makes some students stand up for each negative or positive type of bystander in bullying situations.

As such, this study identifies factors associated with each of the 3 types of bystander behavior (bully followers, outsiders, and defenders of victims) in Korean youth, including important psychosocial and contextual factors based on findings from prior studies such as self-esteem, empathy, social problem solving ability, relationships with friends and teachers, attitude toward bullying, and concern about being bullied in bullying situations, using a cross-sectional design.

The following hypotheses will be tested:

Hypothesis 1: Psychosocial characteristics (self-esteem, empathy, social problem solving ability, and relationships with friends and teachers) will differentially be associated with 3 types of bystander behavior, respectively.

Hypothesis 2: Contextual characteristics (attitude toward bullying and concern about being bullied) will differentially be associated with 3 types of bystander behavior, respectively.

## 2 Methods

### 2.1 Study design

This was a cross-sectional study carried out using a self-report questionnaire to assess 3 types of bystander behavior and related factors.

### 2.2 Participants and data collection procedure

A total of 416 students out of 1065 eligible students attending 1 middle school in Seoul, Korea, voluntarily participated in the study. The list of classes was randomly sorted and then sequentially asked to participate in the study. The first 4 classes agreeing to participate were chosen for the study. Students from 4 classes in each grade (7th to 9th) agreed to participate as a convenient sampling was enrolled in the study. Participants were calculated as 172 using G∗Power 3.1 sample calculation program^[22]^ with significance level of 0.05, power of 95%, medium effect size of 0.15 for linier multiple regression, and 10 independent variables. The number of samples in this study is considered sufficient.

The entire survey took 20 to 30 minutes. Written informed consent was obtained from all participants before inclusion in the study, which had been previously approved by the Sungshin University Institutional Review Board (sswuirb 2012-020). Data were collected from July 8 to 19, 2013 during the final examination period. Each participant completed an in-classroom survey during school hours under the instruction of research assistants. A total of 428 questionnaires were obtained and the final analysis was performed with data from 416 questionnaires as 12 questionnaires had missing items.

### 2.3 Instruments

#### 2.3.1 Demographic characteristics

Students provided demographic information including age, gender, person(s) with whom they live, parental education levels, academic achievement, and socioeconomic status (SES). Academic achievement and SES were based on adolescent self-reporting as low, middle low, middle, middle high, or high. The validity of student-provided information was discussed elsewhere.^[23]^ Family structure was approximated from the information given about the student's living arrangements.

#### 2.3.2 Bystander behaviors

Bystander behavior was measured using an instrument developed and confirmed through the factor analysis from Korean students by Seo^[8]^ based on Salmivalli Participant Role Questionnaire.^[7]^ Students evaluated their behavior as it applied to 32 bullying situation behavior descriptions. From the 32 items, 5 subscales describing tendencies to act as bullies, victims, bully-followers, outsiders, and defenders of victims were formed. This study used scores from a total of 18 items pertaining to bully-followers, outsiders, and defenders of victims. The 6 bully-follower items described tendencies to act in ways that reinforce bullying behavior, such as laughing, coming to see what is happening, and being present during a bullying situation, thus providing an audience for the inciting bully. The 6 items on the outsider scale described “doing nothing” and staying outside of bullying situations. The 6 items on the defender scale described supportive, consoling side-taking with the victim as well as active efforts to make others stop bullying. This self-report measurement was provided on a 5-point Likert scale (0: never, 4: always), and the subscale scores range from 0 to 24. A higher score on each behavior scale meant students exhibit more types of bystander behavior. In this study, Cronbach α values of the bully-followers, outsiders, and defenders were 0.75, 0.82, and 0.78, respectively.

### 2.4 Psychosocial factor

#### 2.4.1 Self-esteem

We measured self-esteem using the Korean version of the Rosenberg Self Esteem Questionnaire.^[24]^ The Rosenberg Self Esteem Scale is the most common measure of self-esteem.^[25]^ It is a 10-item Likert-type scale with items answered on a 4-point scale, from “strongly agree” to “strongly disagree.” Five of the items have positively worded statements and 5 have negatively worded ones, with total scores ranging from 10 to 40 nd lower scores indicating higher self-esteem. The scale measures state self-esteem by asking the respondents to reflect on their current feelings. The Cronbach α in this study was 0.91.

#### 2.4.2 Empathy

The empathy measurement, which was modified for bullying situations by Seo^[8]^ based on Bryant Empathy Index,^[26]^ was 5 items scored on a 5-point scale (1: almost never to 5: almost always). Example items include, “If I see another student suffering from bullying, I also feel the same and become worried,” and “I feel sorry when I see a classmate being bullied.” The scores range from 5 to 25, with higher score indicating greater empathy. The Cronbach α for this measure was 0.82.

#### 2.4.3 Social problem solving ability

Social problem solving ability was measured using the Korean version of the Social Problem-Solving Inventory.^[27]^ This Original version is a 52-item, multidimensional self-report measure of social problem-solving.^[28]^ Items are rated on a Likert scale anchored by 0 (not at all true) and 4 (extremely true). This measure consists of 2 major scales – the Problem Orientation Scale and the Problem-Solving Skills Scale – and 7 subscales. This study used the Rational Problem Solving (RPS) subscales comprised of 20 items to assess the tendency to address daily life problems by applying effective problem-solving practices in a step-by-step, planned, and careful manner. The scores range from 0 to 80, and the Cronbach α for each dimension in this study was 0.92.

#### 2.4.4 Relationship with friends and teachers

From the Personal Relationship Measurement, which was developed by Kim^[29]^ based on the Social Support Survey,^[30]^ 14 items related to friends were selected and modified for school-aged children. Items were measured on a 5-point scale. The Cronbach alpha was 0.96 at the time of development of the instrument and reported as 0.92 in this study. To measure the children's relationships with their teachers, we selected 8 items related to teachers from the School Adjustment Test developed by Im.^[31]^ The items include questions on harmonious relationships between students and teachers, requests for help, responses to scolding, and adequate expressions of positive emotion. The final instrument was a 4-point scale and the Cronbach alpha score was 0.71. Both instruments have been frequently used in studies of school-aged children in South Korea.

### 2.5 Contextual factor

#### 2.5.1 Attitude toward bullying

Attitude toward bullying was measured with the Questionnaire for Korean children developed by Seo^[8]^ based on Salmivalli Attitude toward Bullying Questionnaire.^[3]^ Students were asked to respond on a 5-point scale (0: strongly disagree, 4: strongly agree) the extent to which they agreed or disagreed with 4 statements about bullying. Example items are “Joining in bullying is the wrong thing to do,” and “Bullying may be fun sometimes.” The Cronbach α for this measure was 0.51. The higher a student scored on the scale, the more his/her attitudes were against bullying.

#### 2.5.2 Concerns about being bullied

The concerns about being bullied questionnaire developed by Seo^[8]^ measures the fear of becoming a secondary victim or the loss of popularity in school by being close to a victim. Participants respond on a 5-point Likert scale to 5 total items. Example items are, “I worry that I would also get bullied if I hang out with victims,” and “I think I would be less popular if I helped victims.” The scores range from 5 to 25 with a higher score indicating greater concern about being bullied. The Cronbach α for this measure was 0.89.

### 2.6 Data analysis

Statistical analysis was conducted using PASW software version 20.0. Descriptive statistics were used to examine the demographic characteristics of the study population. *t* tests, ANOVA, and Pearson correlation coefficients were computed to determine differences and assess the relationship between variables and bystander behaviors. Multiple-regression analysis was done to identify variables associated with bystander behaviors. Two-tailed *P* < .05 was defined as statistically significant.

## 3 Results

### 3.1 Study population

Student gender and grade was evenly distributed across the sample. Most students came from nuclear families (88.7%). Both parents had generally completed ≥12 years of education. Most students ranked their perceived SES as middle high (52.4%) and their academic achievement as middle (36.6%) (Table 1).

**Table 1:**
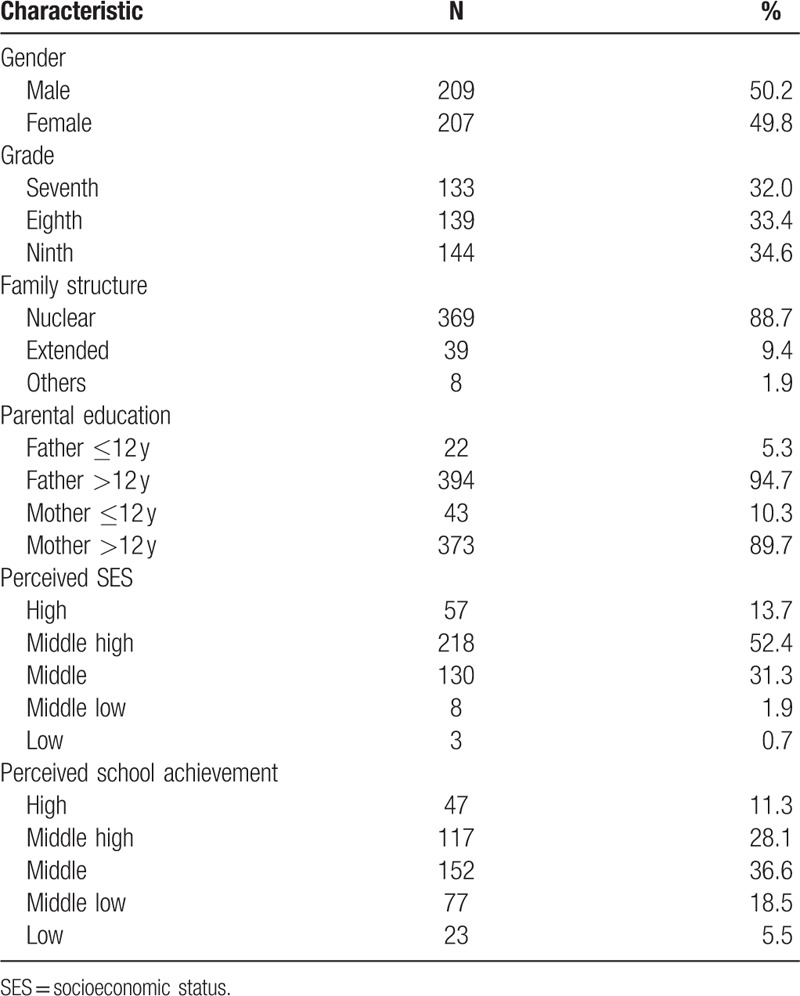
Demographic characteristics of study subjects (N = 416).

### 3.2 Difference in bystander behaviors according to demographic characteristics

There were significant differences in gender (t = 4.634, *P* = .000) and grade (F = 8.52, *P* = .000) among those exhibiting bully-follower behaviors. Perceived academic achievement was significantly different across the 3 types of bystander behaviors (F = 4.515, *P* = .001, F = 3.744, *P* = .005, and F = 3.683, *P* = .006 for bully-followers, outsiders, and defenders, respectively (Table 2).

**Table 2:**
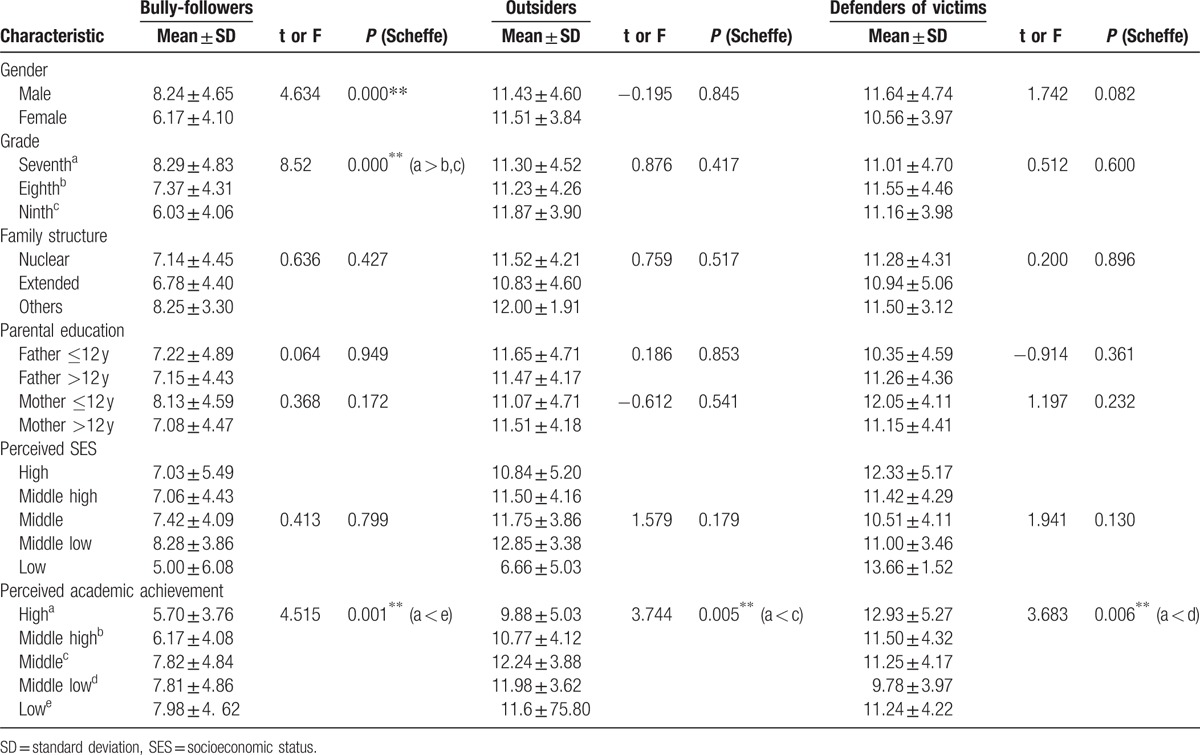
Comparison of bystander’ behaviors according to demographic characteristics (N = 416).

### 3.3 Relationships of major variables with bystander behaviors

All variables in this study were significantly correlated with each type of bystander behavior. Bully-followers, outsiders, and defenders of victims’ were correlated with self-esteem (*r* = −0.144, *P* < .01; *r* = −0.220, *P* < .01; *r* = 0.165, *P* < .01), empathy (*r* = −0.170, *P* < .01; *r* = −0.213, *P* < .01; *r* = 0.255, *P* < .01), social problem solving ability (*r* = −0.138, *P* < .01; *r* = −0.249, *P* < .01; *r* = 0.290, *P* < .01), relationship with friends (*r* = −0.139, *P* < .01; *r* = −0.118, *P* < .01; *r* = 0.108, *P* < .01), relationship with teachers (*r* = −0.305, *P* < .01; *r* = −0.291, *P* < .01; *r* = 0.299, *P* < .01), attitude toward bullying (*r* = −0.346, *P* < .01; *r* = −0.427, *P* < .01; *r* = 0.269, *P* < .01), and concerns about being bullied (*r* = 0.322, *P* < .01; *r* = 0.359, *P* < .01; *r* = −0.254, *P* < .01) (Table 3).

**Table 3:**
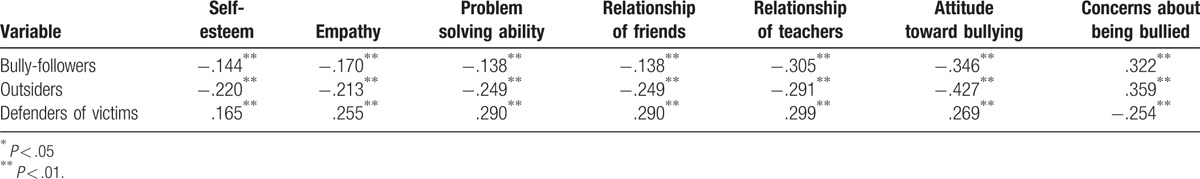
Correlation of major variables and bystander’ behaviors (n = 416).

### 3.4 Variables related to bystander behavior

Multivariate analysis was conducted to assess the independent associations of the tested variables and bystander behaviors. The variables included in the regression model were those found to be significant on *t* tests, ANOVA, and bivariate analysis. Bully-follower behaviors were significantly associated with gender (β = −0.216, *P* = .000), grade (β = −0.187, *P* = .000), academic achievement (β = −0.099, *P* = .049), empathy (β = −0.112, *P* = .033), relationship with teachers (β = −0.152, *P* = .005), attitude toward bullying (β = −0.224, *P* = .000), and concerns about being bullied (β = 0.189, *P* = .000). An adjusted *R*^2^ value of 30% in the regression model highlighted the influence of the included variables on bully-follower behaviors.

Outsider behaviors were significantly associated with empathy (β = −0.189, *P* = .000), relationship with teachers (β = 0.031, *P* = .026), attitude toward bullying (β = −0.334, *P* = .000), and concerns about being bullied (β = 0.206, *P* = .000). Variables in this regression model explained 33% of outsider behavior.

Defender of victim behaviors were significantly associated with self-esteem (β = −0.148, *P* = .044), empathy (β = 0.241, *P* = .000), social problem solving ability (β = 0.219, *P* = .004), relationship with teachers (β = 0.114, *P* = .037), attitude toward bullying (β = 0.182, *P* = .000), and concerns about being bullied (β = −0.231, *P* = .000). The regression model explained 28% of the defender of victim behaviors (Table 4).

**Table 4:**
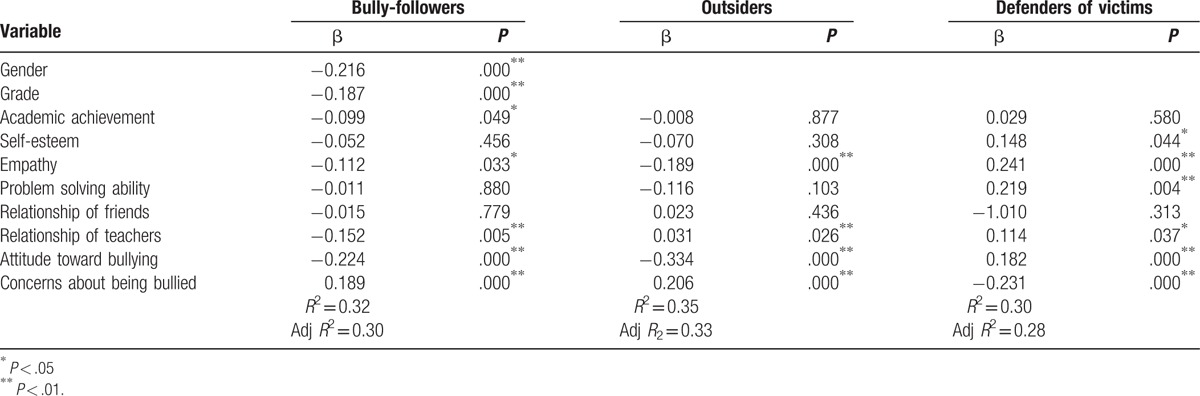
Related factors of bystander behavior.

## 4 Discussion

To better understand how individuals within a school respond as bystanders in bullying situations, it is important to identify factors that influence their behavior. Findings from the present study demonstrated that psychosocial and contextual characteristics differentially associate bystander behavior.

To begin with, demographic factor such as gender, grade, and academic achievement were significantly associated with only bully-follower behavior. Boys were more likely to exhibit bully-follower behavior than girls, a finding that is consistent with previous research.^[8,32]^ Boys tend to believe that aggressive acts are masculine.^[33]^ They have poor judgment with regard to bullying while having a higher tolerance for undesirable behaviors such as bullying, regarding bullying as a joke or a game.^[34]^ Therefore, boys are more likely to be bullies or identify with them. We must design bullying prevention programs that consider these tendencies. Underclassmen and students with low scholastic achievement also tended to exhibit bully-follower behavior. We can infer that younger students do not perceive or judge a bully's behavior to be bad because they are oblivious to the situation or are easily influenced by bullies’ power. In this regard, appropriate bystander behavior must be emphasized to younger students. Students with lower school achievement have a hard time adjusting and being accepted in the school environment in Korea. Therefore, it is possible that they seek acceptance from bullies with power.^[35]^ Adequate comprehensive intervention programs are needed that incorporate an understanding of bullying and the bystander's role and should be provided to underclassmen and students with low scholastic achievement in particular.

Empathy, relationship of teachers, attitudes toward bullying, and concerns about being bullied among variables included in this study were significantly associated with all 3 types of bystanders’ behaviors.

Empathy is one critical individual characteristic that determines whether a child decides to use their social and emotional ability for others^[36]^ and the ability to feel with another and several studies suggest that empathy is related to prosocial and antisocial behaviors.^[37]^ Those with a high level of empathy are able to share the pain inflicted on a victim and experience the emotional fear and torment of the victim's suffering and hence are compelled to inhibit aggressive acts in a bid to reduce their own emotional distress.^[38,39]^ Conversely, their impaired ability to establish the link between their aggressive behavior and others’ suffering prevents bullies from alleviating the latter's distress and discomfort.^[40]^ Youth who engage in bullying also often have low levels of empathy. Thus, empathy is thought to be a key characteristic differentiating bystanders’ role in a bullying situation. Empathy may be a particularly salient characteristic for understanding how students may play role as bystander.^[41]^ Interventions including developing empathy may instill motivation in bystanders to do something to support victims.

Students showing bully-follower and outsider behaviors had poor relationships with teachers whereas students exhibiting defender behaviors did not. Teacher–student interactions and relationships have relevance to general patterns of engagement with students and influence peer systems.^[42]^ When students act for victims, they want the security of knowing that they are protected themselves from their peers and that they will get credit for their good behavior. If the students have a good relationship with teacher, they are more likely to possess those beliefs and thus are more apt to help victims. Teachers are charged with managing the affective or behavioral propensity of their class, and their influence on bullying situations may be through either group or individual interactions with students.^[43]^ Teachers have to support students and foster a class atmosphere that discourages bullying by making ensuring positive interactions and relationships through proactive efforts to give positive feedback about good conduct and consistent discipline. With these efforts teachers will be better able to positively impact their students’ social relationships and the environment in bullying situations and reduce the risk of bullying as a result.

Consistent with previous studies,^[3,44]^ our findings showed that bystander behaviors are associated with attitude toward bullying. Defenders of victims had negative attitudes about bullies and thought bullying was unjust. On the other hand, bully-followers and outsiders viewed bullying as a normal part of adolescence. Even though some researchers insisted that attitude is not always consistently associated with behavior^[45]^ and developing a positive attitude toward victims is not straightforward because bullying situations are based on a complex dynamic,^[46]^ attitude toward bullying might have important implications for bullying interventions. A program that encourages an unfavorable attitude toward bullying is needed for effective antibullying interventions.

Concerns about being bullied were also related to bystander behaviors. Considering the power imbalance involved in a bullying situation, it might be that defending the victim is a retaliatory behavior of those who have previously been bullied when dealing with bullies who are powerful and possibly ruthless in their actions. Many students think that intervening in defense of the victim with low social status puts their reputation at risk.^[7,47]^ Bystanders may avoid taking responsibility out of fear of becoming another target of the bullies, including feelings of fear of retaliation, social disapproval, social blunder, and losing friends.^[48]^ When helping a victim, conflicts between normative justice and disadvantage might arise.^[49]^ Therefore, fostering an environment that does not allow threatening retaliation through consistent enforcement by the teacher and school rules against bullying is needed to encourage students to be active defenders of victims.

On the other hand, self-esteem and social problem solving ability was significantly associated with defenders of victim behaviors. It has been suggested that self-esteem may influence social relationships and human behavior. Higher self-esteem is crucial to adolescents’ social relationships, as it helps them to believe in themselves.^[50]^ In particular, self-esteem in adolescence can influence peer relationships in school.^[51]^ Students who have high self-esteem within the peer context might hold an unshakable attitude in a bullying situation and may help control others in a positive way. Defenders of victim's supportive behaviors may be a manifestation of courage and confidence that are not agitated by the prospect of reprisal in a bullying situation, especially when directed toward weaker peers.

Social problem solving ability was also a very important characteristic among defenders of victims. Social problem solving ability refers to figuring out the most effective ways to deal with stressful or pragmatic situations.^[52]^ Bystanders may fail to take supportive actions because they do not possess effective strategies to counteract bullying.^[53]^ To manage bullying situations or help victims, bystanders should know what to do and how to do it in such situations in order to take action against bullying. For this reason, specific strategies such as ignoring bullies, supporting victims emotionally, telling someone about the bullying, or asking someone for help should be provided to students.

It is encouraging to fine variable such as self-esteem and social problem solving ability to be related to defenders of victim behaviors, since these variables could change bystanders’ behaviors into a positive one. Antibullying programs that include these variables may positively influence and reinforce defenders of victim behaviors, and can be very effective to bullying intervention.

Interestingly, in this study, bully-follower and outsider behaviors were significantly associated with the same factors – empathy, relationship with teachers, attitude toward bullying, and concerns about being bullied. Both the bully-follower's active involvement in bullying and the outsider's desire to avoid the situation allow bullying to persist. The related characteristics of the bully-follower and outsider indicate that an outsider could easily convert to a bully-follower easily in that they both have similar weaknesses. Therefore, we must consider the bully-follower and outsider characteristics in a similar context when designing antibullying interventions.

Our findings have implications in the development of antibullying interventions in the context of bystander's characteristics. Specifically, it may be possible to approach each type of bystander in a personalized way in order to effectively empower bystanders to assume a helpful altruistic role. Our results indicate that an effective intervention should include strategies to foster positive interactions with teachers as reassuring advocates to bystanders, to develop more empathy, and be assured that they themselves will not become victims. Teachers should foster a class atmosphere that aligns with strict school policies against bullying, and work to establish an unfavorable attitude toward bullying through various educational programs.

The bystander's role in bullying situations is important because it has bearing on interventions aimed at reducing bullying.^[13]^ Particularly, as bullying is greatly influenced by collectivism,^[54]^ the bystander has a key role in managing bullying situations. Therefore, it is necessary to design interventions focused on improving bystander behavior.

Our study has some limitations. First, although the study participants were from one middle school in Korea, this was not an epidemiologic sample, possibly limiting the generalizability of the study findings. Second, this study was cross-sectional thus making it impossible to infer causal relationships between bystander behavior and variables examined in this study. Third, the measures of bystander behavior were based on only adolescents’ self-report rather than multiple informants, such as peers and teachers. Despite these limitations, this study clearly shows which factors are associated with bystander behaviors and provide some direction for the development of such preventive and interventional programs.

## 5 Conclusion

This study sought to identify factors associated with each of 3 types of bystander behavior (bully-followers, outsiders, and defenders of victims) in Korean youth. Students who were younger, male, had low school achievement, low empathy, poor relationships with teachers, improper attitude toward bullying, and a high level of concern about being bullied were more likely to be bully-followers. Students having low empathy, poor relationships with teachers, an improper attitude toward bullying, and a high level of concern about being bullied exhibited outsider behaviors including withdrawing from, ignoring, and denying bullying situations. Students who had high self-esteem, empathy, and social problem solving ability, good relationship with teachers, and an unfavorable attitude toward bullying, and less concern about being bullied tended to be defenders of victims.

The results of the present study could contribute to the development of antibullying interventions by highlighting factors significantly associated with specific types of bystander behavior. Further research is needed to confirm and expand on these findings. In addition, future research may seek to evaluate antibullying interventions that are constructed based on findings in this study.

## Acknowledgements

The authors thank the Basic Science Research Program through the National Research Foundation of Korea (NRF) funded by the Ministry of Education, Science and Technology (2012R1A1A2006819) and Sungshin University Research Grant of 2017 for the support.

## References

[1] Olweus D Bullying at school: basic facts and effects of a school based intervention program. J Child Psychol Psychiatry 1994;35:1171–90. 7806605 10.1111/j.1469-7610.1994.tb01229.x

[2] Craig WM Pepler D Atlas R Observations of bullying in the playground and in the classroom. Sch Psychol Int 2000;21:22–36.

[3] Salmivalli C Voeten M Connections between attitudes, group norms and behavior in bullying situations. Int J Behav Dev 2004;28:246–58.

[4] Frey KS Hirschstein MK Edstrom LV Observed reductions in school bullying, nonbullying aggression, and destructive bystander behavior: a longitudinal evaluation. J Educ Psychol 2009;101:466–81.

[5] Juvonen J Graham S Peer Harassment in School: The Plight of the Vulnerable and Victimized. 2001;New York: The Guilford Press, 3–20.

[6] DeRosier ME Cillessen AHN Coie JD Group social context and children's aggressive behavior. Child Dev 1994;65:1068–79. 7956465

[7] Salmivalli C Lagerspetz K Björkqvist K Bullying as a group process: participant roles and their relations to social status within the group. Aggress Behav 1996;22:1–5.

[8] Seo MJ Participation in bullying: bystanders’ characteristics and role behaviors. J Child Stud 2008;29:19–41.

[9] Thornberg R Jungert T Bystander behavior in bullying situations: basic moral sensitivity, moral disengagement and defender self-efficacy. J Adolesc 2013;36:475–83. 23522703 10.1016/j.adolescence.2013.02.003

[10] Kärnä A Voeten M Poskiparta E Vulnerable children in varying classrooms contexts: bystander's behaviors moderate the effects of risk factors on victimization. Merrill-Palmer Quart 2010;56:261–82.

[11] Nocentri A Menesini E Salmivalli C Level and change of bullying behavior during high school: a multilevel growth curve analysis. J Adolesc 2013;36:495–505. 23523327 10.1016/j.adolescence.2013.02.004

[12] Pöyhönen V Juvonen J Salmivalli C Standing up for the victim, siding with the bully or standing by? Bystander responses in bullying situations. Soc Dev 2012;21:722–41.

[13] Salmivalli C Voten M Poskiparta E Bystanders matter: associations between reinforcing, defending, and the frequency of bullying behavior in classrooms. J Clin Child Adolesc Psychol 2011;40:668–76. 21916686 10.1080/15374416.2011.597090

[14] Terranova AM Factors that influence children's responses to peer victimization. Child Youth Care Forum 2009;38:253–71.

[15] Ah YA The mediation effect of self-esteem in the relation between experience of being respected for one's human rights bystanders role in school violence of youths. Correction Welfare Res 2016;40:75–98.

[16] Jung JY Lee SY Oh IS The effectiveness of a school violence bystanders program. J Educ Stud 2013;44:119–43.

[17] Choi S Cho YI Influence of psychological and social factors on bystanders’ roles in school bullying among Korean-American students in the United States. Sch Psychol Int 2013;34:67–81.

[18] Jungert T Piroddi B Thornberg R Early adolescents’ motivations to defend victims in school bullying and their perceptions of student–teacher relationships: a self-determination theory approach. J Adolesc 2016;53:75–90. 27654402 10.1016/j.adolescence.2016.09.001

[19] Dodge KA Lansford JE Burks VS Peer rejection and social information processing factors in the development of aggressive behavior problems in children. Child Dev 2003;74:374–93. 12705561 10.1111/1467-8624.7402004 PMC2764280

[20] Ajzen I The theory of planned behavior. Organizational Behav Hum Decis Process 1991;50:179–211.

[21] Stueve A Dash K O’Donnell L Rethinking the bystander role in school violence prevention. Health Promotion Pract 2006;7:117–24. 10.1177/1524839905278454 16410428

[22] Faul F Erdfelder E Buchner A Statistical power analyses using G^∗^Power 3.1: test for correlation and regression analyses. Behav Res Methods 2009;41:1149–60. 19897823 10.3758/BRM.41.4.1149

[23] Kim YS Boyce WT Koh YJ Time trends trajectories and demographic predictors of bullying: a prospective study in korean adolescents. J Adolesc Health 2009;45:360–7. 19766940 10.1016/j.jadohealth.2009.02.005

[24] Jon B Self-esteem: a test of its measurability. Yonsei Res 1974;11:107–24.

[25] Huang C Dong N Factor structures of the Rosenberg Self-Esteem Scale: a meta-analysis of pattern matrices. Eur J Psychol Assess 2012;28:132–8.

[26] Bryant BK An index of empathy for children and adolescents. Child Dev 1982;53:413–25.

[27] Choi ES A study of the reliability and validity of the social problem solving inventory. Korean J Psychol Soc Issues 2002;21:413–28.

[28] Maydue-Olivares A D’Zurilla TJ A factor-analytic study of the social problem solving inventory: An integration of theory and data. Cognit Ther Res 1996;20:115–33.

[29] Kim JH The relation between daily stress and emotional experience on the adjustment of middle-aged women: impacts of psychological and social resources. J Korean Psychol Assoc 1992;4:54–68.

[30] Sherbourne CD Stewart AL The MOS social support survey. Soc Sci Med 1991;32:705–14. 2035047 10.1016/0277-9536(91)90150-b

[31] Im JS The relationships between dependency and school adjustment of children. Seoul, Korea: Unpublished Master's Thesis, Korea National University of Education; 1993.

[32] Kim YH Han SY Discriminant analysis of bullying participant roles among children. J Child Stud 2011;32:19–41.

[33] Kim EA Lee SY The roles of empathy, self-efficacy, and beliefs in classroom norm in defending behaviors among middle school students. Korean J Dev Psychol 2011;24:59–77.

[34] Hyun JH A study on cognition of bullying by gender. J Korea Jpn Educ 2000;4:133–47.

[35] Moon ES Kim CH A structural analysis of the social and psychological variables influencing adolescents’ school adjustment behaviors. Korean J Educ Psychol 2002;16:219–41.

[36] Björkqvist K Österman K Kaukiainen A Social intelligence-empathy aggression? Aggress Violent Behav 2000;5:191–200.

[37] Gini G Albiero P Benelli B Does empathy predict adolescents’ bullying and defending behavior? Aggress Behav 2007;33:467–76. 17683107 10.1002/ab.20204

[38] Gini G Albiero P Benelli B Determinants of adolescents’ active defending and passive bystanding behavior in bullying. J Adolesc 2008;31:93–105. 17574660 10.1016/j.adolescence.2007.05.002

[39] Wolke D Woods S Bloomfield L The association between direct and relational bullying and behavior problems among primary school children. J Child Psychol Psychiatry 2000;41:989–1002. 11099116

[40] Jolliffe D Farrington DP Examining the relationship between low empathy and bullying. Aggress Behav 2006;32:540–50.

[41] Williford A Boulton AJ Jenson JM Transitions between subclasses of bullying and victimization when entering middle school. Aggress Behav 2014;40:24–41. 24014167 10.1002/ab.21503

[42] Gest SD Rodkin PC Teaching practice and elementary classroom peer ecologies. J Appl Dev Psychol 2011;32:288–96.

[43] Farmer TW Lines MM Hamm JV Revealing the invisible hand: the role of teachers in children's peer experiences. J Appl Dev Psychol 2011;32:247–56.

[44] Rigby K Slee PT Dimensions of interpersonal relation among Australian children and implications for psychological well-being. J Soc Psychol 1993;133:33–43. 8464217 10.1080/00224545.1993.9712116

[45] Augoustinos M Walker I Donaghue N Social Cognition: An Integrated Introduction. 3rd ed. London: Sage; 2014.

[46] Pozzoli T Gini G Active defending and passive bystanding behavior in bullying: the role of personal characteristics and perceived peer pressure. J Abnorm Child Psychol 2010;38:815–27. 20228996 10.1007/s10802-010-9399-9

[47] Seo MJ Exploration on mitigative factors of bullying – focused on analysis by type of outsider. 2006;Busan, Korea: Busan University, Doctoral Dissertation.

[48] Forsberg C Thornberg R Samuelsson M Bystanders to bullying: forth to seventh grade students perspectives on their reactions. Res Paper Educ 2014;29:557–76.

[49] Hazler RJ Bystanders: an overlooked factor in peer on peer abuse. J Professional Couns 1996;11:11–21.

[50] Rosenberg M Society and the Adolescent Self-Image. Pennsylvania, Darby: Diane Publishing Company; 1999.

[51] Fenzel LM Prospective study of changes in global self-worth and strain during the transition to middle school. J Early Adolesc 2000;20:93–116.

[52] D’Zurilla TJ Nezu AM Development and preliminary evaluation of the Social Problem-Solving Inventory. J Consult Clin Psychol 1990;2:156–63.

[53] Lodge J Frydenberg E The role of peer bystanders in school bullying: positive steps toward promoting peaceful schools. Theory Pract 2005;44:329–36.

[54] Kwak KJ Korean society and educational achievement: the flipside of achievement-delinquency among Korean adolescents: Korean Wang-ta: characteristics and prevention program. Korean J Psychol Soc Issues 2008;14:255–72.

